# Mobile Health Apps for the Control and Self-management of Type 2 Diabetes Mellitus: Qualitative Study on Users’ Acceptability and Acceptance

**DOI:** 10.2196/41076

**Published:** 2023-01-24

**Authors:** Marloes Bults, Catharina Margaretha van Leersum, Theodorus Johannes Josef Olthuis, Robin Enya Marije Bekhuis, Marjolein Elisabeth Maria den Ouden

**Affiliations:** 1 Technology, Health & Care Research Group Saxion University of Applied Sciences Enschede Netherlands; 2 Science, Technology, and Policy Studies Faculty of Behavioural, Management and Social Sciences University of Twente Enschede Netherlands; 3 Care & Technology Research Group Regional Community College of Twente Hengelo Netherlands; 4 Department of Smartup Innovation Ziekenhuisgroep Twente Almelo Netherlands

**Keywords:** type 2 diabetes, self-management, mobile health, mHealth, mobile apps, mobile phone, acceptability, acceptance, diabetes

## Abstract

**Background:**

Mobile health apps are promising tools to help patients with type 2 diabetes mellitus (T2DM) improve their health status and thereby achieve diabetes control and self-management. Although there is a wide array of mobile health apps for T2DM available at present, apps are not yet integrated into routine diabetes care. Acceptability and acceptance among patients with T2DM is a major challenge and prerequisite for the successful implementation of apps in diabetes care.

**Objective:**

This study provides an in-depth understanding of the perceptions of patients with T2DM before use (acceptability) and after use (acceptance) regarding 4 different mobile health apps for diabetes control and self-management.

**Methods:**

A descriptive qualitative research design was used in this study. Participants could choose 1 of the 4 selected apps for diabetes control and self-management (ie, Clear.bio in combination with FreeStyle Libre, mySugr, MiGuide, and Selfcare). The selection was based on a systematic analysis of the criteria for (functional) requirements regarding monitoring, data collection, provision of information, coaching, privacy, and security. To explore acceptability, 25 semistructured in-depth interviews were conducted with patients with T2DM before use. This was followed by 4 focus groups to discuss the acceptance after use. The study had a citizen science approach, that is, patients with T2DM collaborated with researchers as coresearchers. All coresearchers actively participated in the preparation of the study, data collection, and data analysis. Data were collected between April and September 2021. Thematic analysis was conducted using a deductive approach using AtlasTi9.

**Results:**

In total, 25 coresearchers with T2DM participated in this study. Of them, 12 coresearchers tested Clear, 5 MiGuide, 4 mySugr, and 4 Selfcare. All coresearchers participated in semistructured interviews, and 18 of them attended focus groups. Personal health was the main driver of app use. Most coresearchers were convinced that a healthy lifestyle would improve blood glucose levels. Although most coresearchers did not expect that they need to put much effort into using the apps, the additional effort to familiarize themselves with the app use was experienced as quite high. None of the coresearchers had a health care professional who provided suggestions on using the apps. Reimbursement from insurance companies and the acceptance of apps for diabetes control and self-management by the health care system were mentioned as important facilitating conditions.

**Conclusions:**

The research showed that mobile health apps provide support for diabetes control and self-management in patients with T2DM. Integrating app use in care as usual and guidelines for health care professionals are recommended. Future research is needed on how to increase the implementation of mobile health apps in current care pathways. In addition, health care professionals need to improve their digital skills, and lifelong learning is recommended.

## Introduction

### Background

The number of patients with type 2 diabetes mellitus (T2DM) is increasing worldwide, creating substantial economic difficulties in many countries, especially in Western Europe [1,2]. In the Netherlands, it is one of the most common chronic diseases, with an expected prevalence of 1.14 million people with T2DM in 2025 and up to 1.33 million people in 2040 [3]. A healthy lifestyle, that is, adherence to regular physical activity and a healthy diet, contribute to the treatment and prevention of T2DM [4-9]. Bassuk and Manson [6] demonstrated that physical activity may contribute to a 30% to 50% reduction in the development of T2DM.

### Apps for Diabetes Control and Self-management

Patients with T2DM require diabetes control and self-management skills to change their lifestyle and adhere to a healthy lifestyle. Self-management has been defined as *“*day-to-day activities or actions an individual must undertake to control or reduce the impact of disease on their health and well-being to prevent further illness*”* [10,11]. Self-monitoring blood glucose levels, food intake, physical activity, and stress can increase the self-management of patients with T2DM. Hence, mobile health apps are promising tools to help patients with T2DM in diabetes education, self-management, and lifestyle modifications to improve their health status and thereby reach diabetes prevention [12-14]. Greenwood et al [13] concluded that the most effective technology-enabled diabetes self-management solutions incorporated 2-way communication, personal data analysis, tailored education, and individualized feedback. The availability of mobile health apps for T2DM has increased significantly in recent years. Although there is a wide array of apps currently available, they are not yet integrated in routine diabetes care. Previous studies have described that end users, staffing, technology, systems, clinical and cultural issues, and costs hinder the acceptance and implementation of mobile health apps for diabetes control and self-management in routine care [15-18].

### Technology Acceptability and Acceptance

The acceptance of apps for diabetes control and self-management among patients with T2DM is a complex process that can vary between different diabetes apps and individuals. Different definitions of acceptability, acceptance, and adoption have been proposed. In this study acceptability is defined as *“*a persons’ perception of a system before use*”* and acceptance as *“*a persons’ perception of the system after use*”* [19]. The Technology Acceptance Model and the Unified Theory of Acceptance and Use of Technology (UTAUT) are widely used technology acceptance models to understand why users accept or reject a specific technology [20,21]. Acceptability and acceptance are key elements of these technology acceptance models. To explain acceptance, UTAUT includes 4 key constructs: performance expectancy (the belief that an app will help improve health performance), effort expectancy (level of ease associated with using an app), social influence (social support), and facilitating conditions (infrastructural support).

### Aim

This study aimed to gain an in-depth understanding of the perceptions of patients with T2DM both before use (ie, acceptability and expectations) and after use (ie, acceptance and actual experiences) regarding 4 different mobile health apps for diabetes control and self-management.

## Methods

### Research Design

This study is based on a qualitative descriptive research design [22]. The methodological orientation underpinning this approach was a naturalistic inquiry [23] to explore the multiple and subjective expectations, perceptions, and actions of patients with T2DM before and after the use of apps for diabetes control and self-management. To explore the acceptability and acceptance of the 4 different apps, 25 semistructured in-depth interviews were conducted, followed by 4 focus group discussions.

This study used the citizen science approach [24]. Patients with T2DM, who were lay people (nonscientists) from the community, collaborated with the researchers as coresearchers in all phases of the study. All coresearchers actively participated in the preparation of the study, data collection, and data analysis. Hence, the coresearchers participated in the development of the interview guide and topic list of the focus group discussion and tested 1 of the 4 apps for diabetes control and self-management. Furthermore, the researchers discussed their perceptions before and after use with other coresearchers. Coresearchers also played an active role as chairs of the focus group discussions, giving feedback on the report, and contributing to interviews for publication as news items in journals and webinars.

### Setting

This study was part of the TOPFIT Citizenlab project. TOPFIT Citizenlab is a 3-year research and innovation program in the eastern part of the Netherlands. Citizens, health care professionals (HCPs), and companies have joined forces with researchers to develop and implement technology for health and well-being.

In this study, 4 apps (ie, Clear.bio in combination with FreeStyle Libre, mySugr, MiGuide, and Selfcare) for diabetes control and self-management were selected. The 4 apps were selected as they were specifically developed for T2DM control and self-management. Furthermore, these 4 apps met mobile health app requirements [25], and providers were willing to participate in citizen science projects. The (functional) requirements were categorized as follows: (1) monitoring (eg, the possibility of monitoring blood glucose levels and different lifestyle factors), (2) data collection and interpretation (eg, visualization of data), (3) provision of information (eg, education regarding healthy lifestyle and diabetes control), (4) coaching (eg, coaching based on behavior change models), and (5) privacy and security (eg, privacy statement, data storage and sharing, and certificates). These (functional) requirements were defined based on different conversations with experts (eg, HCPs involved in diabetes care, IT experts, technology providers, and privacy and security officers).

The Clear.bio app provides insights into a person’s response to nutrition by measuring their blood glucose levels continuously using the FreeStyle Libre flash glucose monitoring system. In addition, Clear is used to monitor food intake, mood, exercise, and sleep. mySugr is an app used to monitor blood glucose levels, food and medication registration, and daily activities. MiGuide is an app focused on healthy lifestyle and behavior changes by coaching patients with T2DM regarding food intake, daily activity, and blood glucose monitoring following a blended care approach. Selfcare is a personal health environment that connects sensors and wearable devices for health and well-being on an independent platform and includes, for example, challenge-based gamification.

### Recruitment Strategy and Sample Size

Coresearchers were recruited via the Dutch Diabetes Association, flyers, and announcements on social and regional media. All those interested in the study were invited to join an introductory webinar. During this webinar, participants digitally met the researchers and representatives of the companies using the 4 apps for diabetes control and self-management. An introduction to the project and the 4 apps was presented, and the participants could ask questions about the project. After the webinar, the participants were asked whether they wanted to participate and, if so, which app they were particularly interested in. Those interested in participation received a letter containing written information about the project and an informed consent form. The informed consent was obtained before the first interview.

Only people who were diagnosed with T2DM and had the intention to work together with the researchers, as coresearchers, were included in this citizen science project. Furthermore, a minimal level of digital skills and having a mobile phone was required to participate in this study. The coresearchers had no previous experience with any of the apps. The time since the diagnosis was not included in the inclusion or exclusion criteria. According to the guidelines for qualitative research, we attempted to include a minimum of 20 coresearchers [26].

### Data Collection

Data were collected between April and September 2021 through semistructured interviews and focus groups. All coresearchers performed a short exercise in preparation for the interviews. They received 2 pictures of flowers with empty leaves [27]. They were asked to share their motivations to participate in the study, especially regarding the app and their role as coresearchers, and write them down on the leaves of the flower (Multimedia Appendix 1). Semistructured in-depth interviews were conducted via telephone calls. The calls lasted between 30 and 60 minutes. The interviews were audio recorded and the researchers took notes during the interviews. Three researchers conducted interviews (MB, CMvL, and TJJO). The interviews focused on the perceptions and expectations of the coresearchers, considering their choice to test and use one of the apps for diabetes control and self-management (Multimedia Appendix 2). In addition, one question derived from the Personal Innovativeness Scale was included in the topic list [28].

The interviews were followed up by 4 focus group discussions with the same coresearchers 4 months after testing the app of their choice. The focus groups took place at a central location in one of the affiliated research centers to minimize the burden and travel distance to participate in the focus groups. Three researchers (MB, CMvL, and TJJO) and 2 coresearchers arranged and chaired the focus group discussions. The focus group discussions lasted for approximately 90 minutes. Audio recordings were made of the focus groups, and a researcher (CMvL) observed and made notes on the discussions and interactions between the participants. Two focus groups were organized to discuss user acceptance of Clear, one about MiGuide, and in one focus group, coresearchers who tested mySugr and Selfcare were combined (owing to the smaller number of participants). The topic list for the focus group was prepared in collaboration with several other coresearchers. The topics included perceptions, experiences, reflections on the expectations, role of HCPs, and the information provided by the apps for diabetes control and self-management (Multimedia Appendix 3).

### Data Analysis

Data from flower associations were analyzed and described in infographics. The data from the semistructured in-depth interviews and focus groups were combined and analyzed following the same steps. First, the interviews were transcribed verbatim, and extensive observation notes from focus group discussions were used. On the basis of the coding process, the notes of the focus groups were complemented with verbatim transcriptions where needed. Measures were taken to avoid cross-contamination of data: that is, by a clear overview of which coresearcher used which app, checks in the follow-up questions to ensure which app they were talking about, adding researchers’ notes to the transcripts (link with app), and a summary was sent to all participants linking the quotes and results to the different apps (member check). All data were analyzed using a deductive approach [29]. This deductive approach followed the elements of the UTAUT model (Multimedia Appendix 4). The transcripts and observation notes were read, and codes were assigned to specific passages. Three researchers (MB, CMvL, and TJJO) coded the data and compared them. The findings were discussed iteratively with the project team during weekly meetings. The researchers used the software package AtlasTi9 to analyze the data. Data saturation was achieved when no new themes emerged in the transcripts.

### Trustworthiness

Credibility was established through several procedures [23]. Method triangulation (ie, interviews and focus groups) was conducted to increase the credibility of the data and study. In addition, audio recordings, notes, and observations were combined to gain in-depth insight into the perceptions of patients with T2DM before and after the use of mobile health apps. Investigator triangulation was achieved as several researchers designed the study, read the transcripts and notes, analyzed the data, and compared the findings. Furthermore, the research team consisted of professional researchers from different research institutes and patients with T2DM as coresearchers. Peer debriefing took place at weekly meetings with the project team, where both scientific and organizational aspects were discussed. The summarizing document of the project was shared with all the coresearchers and app developers as part of the member check.

A thick description was developed for transferability, which included recruitment, coresearchers’ selection, data collection, and data analysis. This citizen science approach to testing apps for diabetes control and self-management is a transferable method to be used in other settings and development contexts.

### Ethics Approval

Ethical review and approval were obtained from the Ethics Review Committee of the University of Twente (210043). The coresearchers provided written informed consent and were informed about their right to withdraw at any time. Data were anonymized, and data confidentiality was maintained.

## Results

### Demographic Characteristics of the Coresearchers

In total, 25 coresearchers with T2DM participated in this study. Overall, 48% (12/25) of the coresearchers tested Clear, 20% (5/25) MiGuide, 16% (4/25) mySugr, and 16% (4/25) Selfcare. All coresearchers participated in semistructured in-depth interviews, and 18 of them attended focus groups. Of the coresearchers, 52% (13/25) were female, and 48% (12/25) were male (Table 1). The mean age of the coresearchers was 63 (SD 7.6, range 47-77) years. More than half (14/25, 56%) of the coresearchers had been diagnosed with T2DM ≥10 years ago, and 44% (11/25) had been diagnosed with T2DM ≤10 years ago. Most coresearchers used oral medication (14/25, 56%) or insulin (9/25, 36%).

**Table 1:** Demographic characteristics of the coresearchers (N=25).

Characteristics		Value, n (%)
**Sex**		
	Male	12 (48)
	Female	13 (52)
	Intersex	0 (0)
**Age range (years)**		
	40-49	1 (4)
	50-59	8 (32)
	60-69	10 (40)
	70-79	6 (24)
**Disease duration (years)**		
	<5	4 (16)
	5-9	7 (28)
	10-14	8 (32)
	15-20	5 (20)
	>20	1 (4)
**Type of medication**		
	None	2 (8)
	Oral	14 (56)
	Insulin	9 (36)

### UTAUT Constructs

The results are based on the constructs of the UTAUT model, describing performance expectancy, effort expectancy, social influence, facilitating conditions, anxiety, and trust in data security and knowledge. General findings and quotes from the coresearchers were acquired during the interviews and focus groups. Within each construct of the UTAUT model, perceptions regarding expectations before use (ie, acceptability) are followed by actual experiences after use of the app (ie, acceptance) for diabetes control and self-management.

### Performance Expectancy

Personal health was the main driver of app use. This was already visible in the flower associations. There were quite some written comments, such as “losing weight,” “less medication,” “understand the effect of nutrition,” and “less stress.” All coresearchers had their personal goals and believed that the apps would help reach these health-related goals. The goals ranged from reaching a stable blood glucose level to losing weight, more healthy diets, or exercising more often. A small number of coresearchers had the expectation of lowering the need for medication. Overall, all the coresearchers expected that they would learn more about the influence of nutrition:

I can imagine that one kind of carbohydrate will have a different effect on my body compared to another kind of carbohydrate, for example pasta or bread. This does not mean I will eat all these different carbohydrates to test the effect on my body, but I would like to know on which kind of nutrition I react best or worst. Also, the severity of the reaction and the moment I will feel some effect on my body is interesting to get more knowledge on.

Clear coresearcher

Most coresearchers were convinced that a healthy lifestyle would improve blood glucose levels. This was the main finding after the testing period. They mentioned nutrition, exercise, and stress as having an impact on their lifestyle. “It would be nice to have this overview in the app, to see when you did a lot and when you need to work a bit more on your lifestyle” [MiGuide coresearcher]. Coresearchers wanted insight into their lifestyles to find a balance in their lives. These apps provided helpful insights. However, some coresearchers asked the companies for help, and MiGuide coresearchers all used the app in consultation with a lifestyle coach. The lifestyle coach was a valuable addition to understanding and adhering to advice based on the data from the app.

Continuous monitoring is especially a performance expectation of coresearchers who tested the Clear app. “It will probably help if the sensor just measures, that is more accurate than the measurements I will do myself and it might show how my glucose level is actually evolving during the day” [Clear coresearcher]. However, coresearchers also expected that the use of apps might give them knowledge about their disease and how the disease influences them:

I hope the app will give me a vision on diabetes and how I personally can control the disease. For example, if I eat this, I will know what happens with my blood glucose level, a kind of self-consciousness.

MiGuide coresearcher

They expected the app will “save my choices. There will be a log of everything I eat and how much exercises I performed, which makes me possibly more conscious of making choices” [mySugr coresearcher]. After the test period, most coresearchers confirmed that the apps influenced their lifestyle, but the extent was debatable. Some agreed that “the app was a real ‘eyeopener’ for me” [Clear coresearcher], but others thought that the app just showed them the obvious or was not accurate enough. The impact of the test period ranged from lowering medication and changing the entire diet to taking some advice into account.

### Effort Expectancy

In general, most coresearchers did not expect that they need to put much effort into using the apps. For example, coresearchers who measured their blood glucose levels expressed that the option for more continuous measurements was desirable and that the transition would be small: “I do not like the fingerstick, and with the sensor that will not be necessary” [Clear coresearcher]. Comparing this expectation with the actual experience, coresearchers stated that it was amazing how much data were available and how easily this could be visualized in graphs. The Selfcare app also provided clear visualizations: “the logged data was always visible and visualized in very pretty graphs” [Selfcare coresearcher]. A disadvantage experienced by a Clear coresearcher was that she sometimes forgot to upload data from her sensor to her phone in time. The sensor could only save the last 8 hours of the measurements; therefore, the graphs had some gaps.

Before using the apps, coresearchers wrote in the flower associations that the apps might minimize efforts to maintain a healthy lifestyle. They expected that apps would provide suggestions or directions for action, mainly focusing on nutrition and exercise. This supports coresearchers to keep a grip on their own lives and understand the impact of external factors:

Currently I must figure everything out by myself. When I can test and note everything the app will give me the required feedback to stay on the right track.

Clear coresearcher

The positive experiences of using the app outweigh the additional effort required to use the app. “When you reach a certain success, think about a different lifestyle and gain more knowledge, that is worth testing it” [mySugr coresearcher]. However, based on the experiences during the test period, the additional effort to familiarize themselves with app use was quite high. For all 4 apps, the coresearchers had to keep track and log many details, such as exercise and food intake. As one of the Selfcare coresearchers described:

It was a lot of work to log everything every day, this was a disadvantage of the app. You had to log everything yourself and it is easy to make mistakes.

### Social Influence

None of the coresearchers had a HCP who provided suggestions on using apps. They visited their HCP regularly, but “I visit my general practitioner twice a year, we discuss the blood test results, but it is always a snapshot when they measure the blood glucose levels” [Clear coresearchers]. Although they obtained some knowledge from regular visits, most coresearchers expressed their desire to gain more knowledge about their own bodies and diabetes:

We [diabetes care specialist and patient] discuss the blood test and then it is always the same: “it looks very good, please continue,” but this is not enough.

mySugr coresearcher

The coresearchers who tested the MiGuide app were also supported by a lifestyle coach. The combination of an app and a coach is a positive experience. The coach has a positive influence on coresearchers. She did not forbid the coresearchers anything, but “she holds a mirror and then you can understand yourself what is wrong.” In addition, regular appointments with lifestyle coaches have a positive social influence on adherence to lifestyle changes. All other coresearchers expressed a desire to have such a relationship with HCPs:

The health care professional need to play an important role in our care with the Clear app as daily support. Starting with such an app without the assistance of a health care professional could cause a lot of confusion. Especially someone who is ‘new’ to diabetes can benefit a lot and find its way toward a healthy life pattern.

Clear coresearcher

According to coresearchers, it is simple to monitor, show, and share data with HCPs.

Next to HCPs, social networks (ie, family and friends) play an important role in managing T2DM. Before the start of the test period, almost all the coresearchers felt supported or strengthened by their close relatives. With the challenges provided in the Selfcare app, the coresearchers received support from their relatives. For example, the entire family participated in the “wholegrain-challenge.” In contrast, they sometimes felt misunderstood regarding specific lifestyle choices, for example, not wanting to have a piece of pie during a party. There was one coresearcher, who was going to test Clear, who told us, “My wife will not be interested. She does not want to know about all the things I can do and sees it as a waste of time.” At the start of the study, this complaint was only mentioned one partner. During the test period, more partners complained about the effort needed and “he complained about the amount of time I was using the app” [Clear coresearcher].

### Facilitating Conditions

An issue often mentioned during the interviews, focus groups, and flower associations are the reimbursement options of technologies for self-management and control of people with T2DM.

I mean, normally I will not receive any reimbursement. If I want to perform a fingerstick blood glucose test, I must pay for it.

Clear coresearcher

After the test period, all coresearchers expressed the need for this type of technology to assist people with T2DM and the need for reimbursement. They could test the products now for free, and some agreed that they would pay for it, but they acknowledged that many others would not have the possibility to pay for the technologies themselves.

Another necessary facilitating condition according to the coresearchers was the acceptance of the apps for diabetes control and self-management by the health care system. This could include all the different HCPs involved in diabetes care, as well as improving or changing the standards and protocols on which HCPs base their treatment:

Nowadays they search for a treatment by adding or lowering the number of pills. I am not in favor of such an approach, if it is needed and there is no other option it is ok, but it should be the last resort.

Selfcare coresearcher

If the health care system could facilitate professionals to support diabetes treatment by technology, it would be beneficial for people with T2DM.

### Anxiety

Most of the coresearchers participated in this study because of their interest in technology. None of them expressed any feelings of anxiety regarding technology. However, they were anxious that this technology would not be available to them or would be too expensive for most people with T2DM. Another link with anxiety is their distrust in developers because they have no idea how to live with diabetes. They feel that the technology should be developed more specifically to their needs to assist them in reaching their personal goals of living with diabetes. This might also increase new users’ trust in technology:

I want to experience the technology. Tell the developers about my experience and help them to define which elements works and where they need to improve the app. This is needed to make it future-proof for everyone living with diabetes.

Selfcare coresearcher

One element of the Clear app that raised anxiety was the sensor:

I had to apply the sensor on my arm without help... I left it on my table for two weeks, I was too anxious.

More assistance is required in the first phase of technological use. Finally, all coresearchers applied the sensors themselves or with the help of a family member. Asking for help was an important barrier.

### Trust in Data Security

There were no concerns regarding the data security. All coresearchers expected that the data would be stored safely by companies. In addition, if they would share data (in the future) with their HCPs, the coresearchers expected that the information in the app would be treated with strict confidentiality. Furthermore, all coresearchers had a lot of trust in technology, in general:

I try to know everything about new technologies or other assistive tools for diabetics. I dive into the material and believe that it might improve my life or make it easier.

MiGuide coresearcher

All coresearchers were interested in technology, and most of them performed their own searches on newly available technologies for diabetes:

When a new app crosses my path, I will try to see how useful it is. I would describe myself as that kind of person.

Clear coresearcher

Although most of them were frontrunners in trying out the technology, some coresearchers were more hesitant and curious about experiencing apps. Although most of these more hesitant coresearchers had a difficult start and needed more help from the companies to use the app, they were enthusiastic about the results after using the app, and 2 bought an actual subscription to be able to continue using it after the research period.

### Knowledge

Half of our coresearchers’ group knew apps to support them in their lives with diabetes. Some of them had already tried several apps or were still using them at the start of the study. They used the apps to improve their knowledge about their own body, in addition to acquiring knowledge by reading magazines or searching the internet. However, most coresearchers mentioned that they still struggled to cope with the disease:

I know a lot and learned a lot myself, but I do not understand why it is not working for me. I have tried a lot, but need more help from external factors.

Clear coresearchers

As every person with T2DM is different, they expressed a desire for more personalized care and the integration of apps. Some also mentioned “unique knowledge” in flower associations.

Another aspect of the knowledge discussed after the test period was the need for more information on how to use the app. Coresearchers needed more assistance, especially at the beginning. Where some tried and figured out themselves, “during the course of the test period I scrolled more and more through the app and could use more and more elements of the app” [Selfcare coresearcher], others got lost and confused, “I was completely lost when the scores of my previous meal showed” [Clear coresearcher], and others asked for help, “I contacted the MiGuide developers and they really quickly helped me with everything” [MiGuide coresearcher]. Overall, all coresearchers agreed that more knowledge of apps for diabetes control and self-management was required before the test period and maybe already at an earlier phase in their diabetes trajectory. Their idea was to disseminate the knowledge gained about these apps among HCPs and associations, such as the Dutch Diabetes Association, and to try to reach all people who have recently been diagnosed with T2DM through these channels.

## Discussion

### Summary of Findings

This study provided an in-depth understanding of both the perceptions of patients with T2DM before use (acceptability) and the perceptions of patients with T2DM after use (acceptance) regarding 4 different mobile health apps for diabetes control and self-management. Personal health was the main driver of app use. Most coresearchers were convinced that a healthy lifestyle would improve blood glucose levels. The performance expectation among the coresearchers when using the apps was high. This mainly concerned the expectation that the app would have a positive influence on their health, diabetes control, and self-management by acquiring knowledge and gaining insight into blood glucose levels in relation to diet and exercise. Although most coresearchers did not expect to put much effort into using the apps, the additional effort to familiarize themselves with the app use was quite high. None of the coresearchers had a HCP who provided suggestions on using the apps. One of the reasons might be that mobile health apps are not yet part of the practical guidelines and protocols. When coresearchers are guided by a lifestyle coach when using the apps for diabetes control and self-management, the discipline of participants in pursuing a healthy lifestyle seems to increase. Coresearchers prefer more information about mobile health apps for diabetes control and self-management and how to use these apps. Reimbursement from insurance companies and acceptance of apps for diabetes control and self-management by the health care system were mentioned as important facilitating conditions.

### Reflection With the Literature

The degree of acceptability and acceptance of mobile health apps for the control and self-management of T2DM can vary per app and per patient, as shown in this study. This variety was related to the coresearchers’ personal characteristics, preferences, needs, and experiences. In this study, 96% (24/25) of the participants were aged ≥50 years. Previous research has shown that age is associated with both the intention to use (mobile health) apps and performance expectancy is moderated by age [30]. Hence, future studies should investigate whether similar results are observed in younger patients with T2DM. In addition, apps with functionalities that can adapt to personal preferences and changes in consumer demands are more likely to be used continuously, thereby maintaining positive behavior [31]. This is in line with the recommendations of coresearchers to include personal preferences (settings) in mobile health apps and to receive personalized feedback.

Effort expectancy is one of the main drivers of technology use (eg, apps) [21]. Beforehand, coresearchers did not expect that the use of apps would take a lot of time. In practice, however, coresearchers had to understand the app and thereafter track and log data such as food intake and exercise. Relatives and family members also noticed investment in time. Relatives were usually closely involved in the lives of the coresearchers and the impact of diabetes on their lives, but they were also regularly critical of the time it took to process all data in the app. Hence, realistic information should be provided to patients with T2DM and their relatives to facilitate the long-term use of apps. Especially for patients with minimal digital skills, instruction and coaching of in-app use is of utmost importance [32].

Another barrier was trust in the app developers. Coresearchers have stated that the needs and wishes of patients are not always taken into account when developing apps. The positive effects of mobile health apps for diabetes self-management are maximized through the integration of more comprehensive functionalities, input from patients and professionals, and evidence-based design [33,34].

Smartphones can facilitate communications between patients and caregivers and customize health monitoring for individual patients. Hence, smartphones are uniquely positioned to enable patients to support their daily diabetes self-management [35]. However, HCPs rarely use the data collected by patients to adjust for the treatment of T2DM. Alaslawi et al [36] conducted a review and concluded that HCPs remained hesitant to use diabetes self-management apps. HCPs play an important role in both treatment adherence and long-term health outcomes. Ashrafzadeh and Hamdy [17] showed that patient-professional interactions are essential for improving health outcomes and preventing long-term complications in patients with T2DM. In addition, patients with a higher frequency of patient-physician meetings achieved their hemoglobin A1c, blood pressure, and cholesterol level goals faster and had higher success rates compared with patients who had less frequent contact with their general practitioner [37,38]. Finally, mobile health interventions can change hemoglobin A_1c_–levels more often in patients with T2DM and type 1 diabetes mellitus compared with patients receiving care as usual [39].

Lack of reimbursement has been mentioned as one of the main barriers to using apps for diabetes control and self-management. Hence, reimbursement of apps (eg, by health insurance companies) may have a positive effect on the acceptance and implementation of apps, as well as on health outcomes. To date, there are no or minimal reimbursement options in the Netherlands for apps or technological equipment if patients with T2DM are not insulin dependent. Financial issues in terms of reimbursement have been described as a major challenge in the adoption of digital health for diabetes care [40].

Patients with T2DM struggle to select relevant apps based on their personal preferences and needs. They need structured information and instructions to guide them from their first use. In addition, they prefer the integration of different apps and functionalities to limit the use of multiple apps side by side. Ideally, such apps will be compatible with electronic health records and remote data sharing when adjustments in diabetes care are required [17].

### Strengths and Limitations

The strengths of this study are its collaboration with coresearchers (ie, experts in their disease). All coresearchers actively participated in the preparation of the study, data collection, and data analysis as citizen scientists who were enthusiastic about participating in this study and being coresearchers. They showed interest in the apps selected from different manufacturers. The coresearchers in this study all had years of experience with T2DM. They also had extensive experience collaborating with various professionals in the field of T2DM. Citizen science can be used to exploit existing experiences and ideas. Another strength is that coresearchers were free to choose 1 of the 4 selected apps that matched their personal preferences. Furthermore, this study provides an in-depth understanding of the perceptions of patients with T2DM before use (acceptability) and after use (acceptance). The limitations of this study include its characteristics of the study population. Coresearchers are more likely to have higher digital literacy and motivation compared with other patients with T2DM (response bias). All participants were interested in technology in relation to T2DM and had a higher-than-average level of education. There was a certain degree of acceptance and adoption of technology among the respondents, with all having a great discipline in the field of self-management in relation to T2DM. Most of the coresearchers were early adopters of technology and had extensive experience using different technologies for diabetes control and self-management.

### Recommendations

Research has shown that mobile health apps provide support for diabetes control and self-management in patients with T2DM. Coresearchers have suggested that the benefits are higher when app use is combined with support from an HCP. The preferred functionalities of apps for T2DM control and self-management differ among coresearchers. Therefore, it is important that functionalities and visualizations in apps can be customized to personal preferences and needs. Developers should collaborate with patients with T2DM and experts during the development to optimize apps, for example, reducing the number of actions to enter data. Integration of app use in care as usual and guidelines for HCPs are therefore recommended. In particular, HCPs use the data obtained from patients for follow-up treatment. Future research is needed on how to increase technology implementation in the current care pathways. In addition, HCPs need to improve their digital skills, and lifelong learning is recommended.

### Conclusions

Personal health was the main driver to start using apps to improve diabetes control and self-management. Before using the apps, coresearchers expected limited effort to use the apps, did not feel anxious and were not concerned about data security. However, after the initial phase, coresearchers needed more guidance and information on how to use the apps, and based on coresearchers’ perceptions, both HCPs and relatives played an important role in app use and compliance. Acceptance and adoption of apps can increase if users can personalize functionalities, reimbursements are available, the number of data entry operations is reduced, and if different functionalities are combined in one app.

## Appendix

Multimedia Appendix 1 Example of flower associations.

Multimedia Appendix 2 Topic list semistructured in-depth interviews.

Multimedia Appendix 3 Topic list focus groups.

Multimedia Appendix 4 Coding tree.

## Abbreviations

HCP: health care professional
T2DM: type 2 diabetes mellitus
UTAUT: Unified Theory of Acceptance and Use of Technology
