# Supplementary material for: Mobile Health Apps for the Control and Self-management of Type 2 Diabetes Mellitus: Qualitative Study on Users’ Acceptability and Acceptance
Source: JMIR Diabetes. 2023 Jan 24;8:e41076. doi: 10.2196/41076 (PMC9947812; doi:10.2196/41076)

**Multimedia Appendix 1. Example of flower associations**

In preparation of the interview on [date], I would like to ask you to make a flower association. This is a creative way to think about specific topics.

[Name of the app] & Me as citizen scientist

- What do you think about when you read these terms?
- What comes up to your mind?
- Advantages or disadvantages?
- Questions about this topic?

I'm curious about that!

I would like to ask you to write or draw thoughts. You are free to interpret it in your own way. You may add leaves, make drawings, write around the flower. Everything is possible.

Could you please take a picture of this and send it to me?

Yours sincerely,


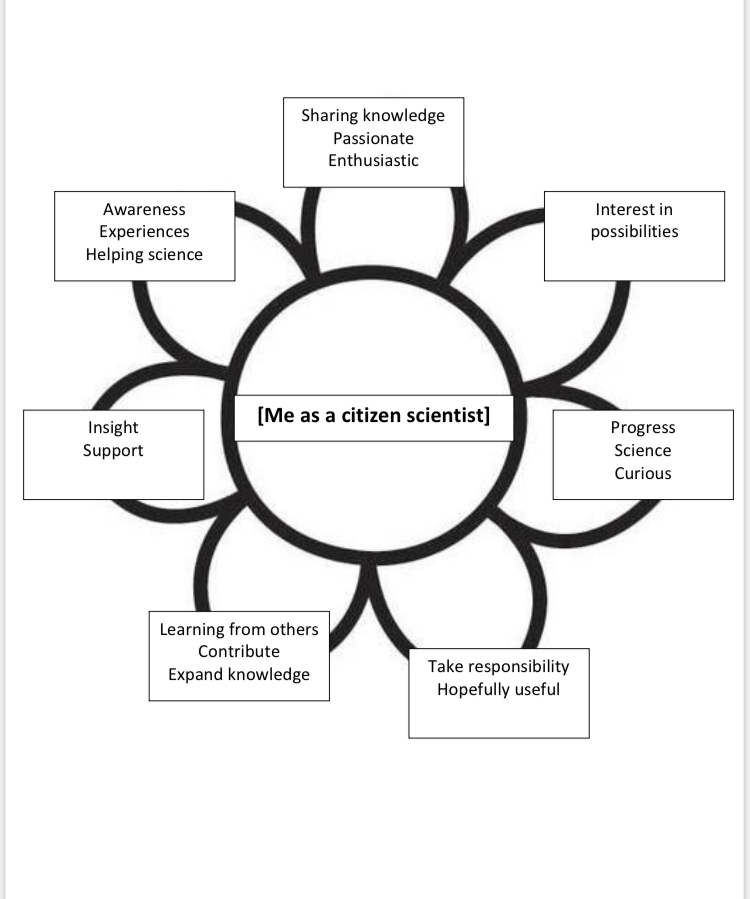
[name of researcher and contact details]


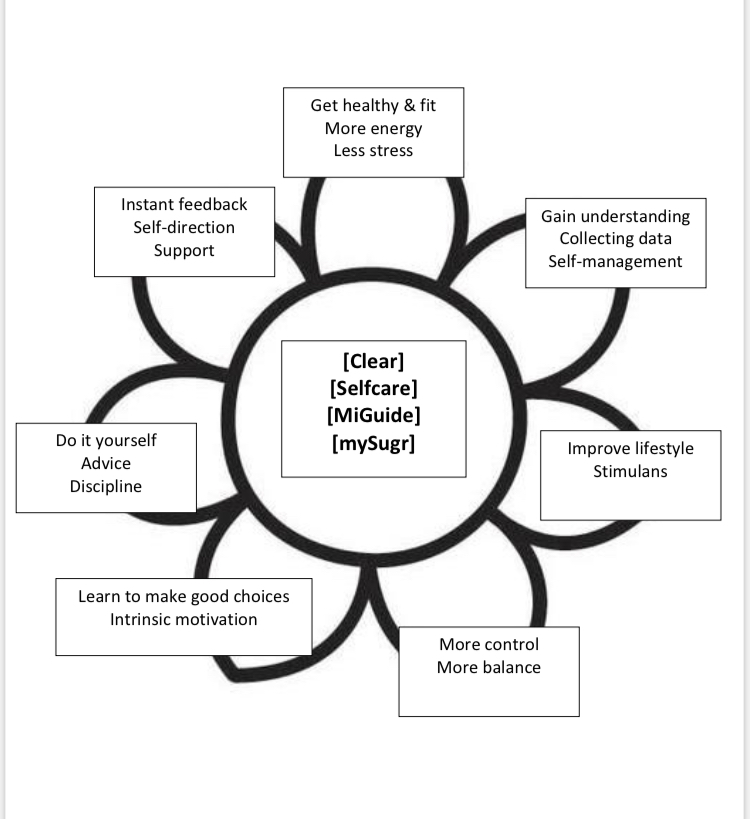

Supplement: Multimedia Appendix 1 [file diabetes_v8i1e41076_app1.docx]
