# Supplementary material for: Mobile Health Apps for the Control and Self-management of Type 2 Diabetes Mellitus: Qualitative Study on Users’ Acceptability and Acceptance
Source: JMIR Diabetes. 2023 Jan 24;8:e41076. doi: 10.2196/41076 (PMC9947812; doi:10.2196/41076)
Supplement: Multimedia Appendix 2 [file diabetes_v8i1e41076_app2.docx]

**Multimedia Appendix 2. Topic list semi-structured in-depth interviews**

1. **Living with diabetes**

- Can you briefly tell us about yourself?
- Age
- Education level / profession
- Nationality
- Living situation / family composition
- Social network
- Health situation
- Informal care

1. **Defining own health**

- What is important for your health?
- What is your definition of health?
- How would you rate your own health?
- What would contribute to improving your health, what do you need?

1. **Daily life & influence of diabetes**

- What do you do during a normal day/week?
- What do you like to do in your life?
- How long have you been diagnosed/how did it start?
- How do you experience the influence of diabetes on your daily life?
- How does diabetes affects your daily life?
- How do you experience having diabetes?

1. **Reason for participating in the study**

- What was the reason/motivation to participate in this study?
- How did you receive information about this study (i.e. door-to-door newspapers, social media or previous participation)?
- Could you explain what you described at the flower association under “Me as a citizen scientist/co-researcher”?

1. **Reason/motivation to try mobile health application?**

- What is the reason/motivation that you want to try an application?
- Which of the following statements do you agree with and why?
- I am usually ahead of the rest and often have innovative ideas. I am always the first to use a digital tool (innovator)
- I like to try new things and experiment with digital tools (early adopter)
- I don’t use digital tools, until I know it is useful. I will then also recommend it to others (early majority)
- I don’t use digital tools until a lot of other people use it and I see it is convenient and useful (late majority)
- I don’t use digital tools quickly, because I like to stick with what I already know and use (laggards)

1. **Goals and expectations mobile health application**

- Which mobile health applications did you choose?
- Where you already familiar with this mobile health application?
- Why did you choose for this mobile health application?
  - What were the main reasons?
- Which factors played a role in the choice for this mobile health application (i.e. information manufacturer, social environment, available reviews, number of users)?
- In the flower association, you described that [mention words co-researcher described on the flower association]?
  - Can you explain this?
- What are your expectation of this mobile health application?
  - How should the use of this mobile health application influence your daily life?
- Which goals do you want to reach by using this mobile health application?
- Do you think the use of mobile health applications influences your diabetes self-management and control?

1. **Actual use**

- Did you already start to use this mobile health application?
- What is the frequency of use of this mobile health application (daily/weekly)?
- How do you experience the use of this mobile health application at the moment?
- Which functionalities are useful and why?
  - What are the advantages of using this mobile health application?
- Which functionalities are not useful and why?
  - What are the disadvantages of using this mobile health application?

1. **Valorisation & information**

- Which information is needed to use this mobile health application for the first time?
- Which knowledge and skills are needed to use this mobile health application?
- Do you know how to interpret the available data?

1. **Experiences with other digital applications**

- Do you use also other digital applications for diabetes self-management and control specifically?
  - If yes, which digital applications and why?
  - If no, why not?
- Do you use also other digital applications for active and health lifestyle?
  - If yes, which digital applications and why (for example Fitbits, Runkeeper)?
  - If no, why not?
- What helps you to use digital applications for a longer period over time?

1. **Social influence**

- Do other patients with type 2 diabetes in your social network make use of mobile health applications?
- Do you experience social pressure or social support from your family member, partner, friends?
  - How do you think your social network can support you in using mobile health applications for diabetes self-management and control?
  - What kind of support do you need?
- Did you receive any information from your healthcare professional (i.e. general practitioner) regarding the availability of mobile health applications for diabetes self-management and control?
  - If yes, which information did your receive from which healthcare professional?
  - If no, did you want to receive information from your healthcare professional?
- Do you think making use of mobile health applications would change the patient-professional relationship? If yes, can you explain this?

1. **Influence on daily life**

- What are your main goals regarding diabetes self-management and control / healthy and active lifestyle?
- Do you think the use of this mobile health applications helps to improve diabetes self-management and control?
- Do you think the use of this mobile health applications helps to improve a health and active lifestyle?

1. **Sharing data**

- Are you willing to share the data you collect by using the app?
  - If yes, with whom?
- Do you expect to share the data with your healthcare professional / general practitioner?
- Does your healthcare professional / general practitioner know you make use of this mobile health application?
