# Supplementary material for: Mobile Health Apps for the Control and Self-management of Type 2 Diabetes Mellitus: Qualitative Study on Users’ Acceptability and Acceptance
Source: JMIR Diabetes. 2023 Jan 24;8:e41076. doi: 10.2196/41076 (PMC9947812; doi:10.2196/41076)
Supplement: Multimedia Appendix 3 [file diabetes_v8i1e41076_app3.docx]

**Multimedia Appendix 3. Topic list focus groups**

1. Experiences
   1. Mobile health application
   2. Pleasant/unpleasant
   3. Advantages/disadvantages
   4. Problems
2. Daily life
   1. Level of blood glucose
   2. Exercise
   3. Diet
   4. Stress
3. Actual use
   1. Frequency of use (daily/weekly)
   2. Involvement of social network
4. Changes
   1. Diabetes self-management and control
   2. Healthy and active lifestyle
5. Adoption
   1. Intention to use over a longer period of time
6. Healthcare professionals
   1. Healthcare professionals informed about use of mobile health application
   2. Reactions of healthcare professionals
      1. Regarding mobile health application
      2. Regarding use
      3. Need for more information
7. Support: medical/technical/daily life
   1. Offered
   2. Desirable
8. Information & Education regarding app (specifically) & technology (in general)
   1. Experiences
   2. Wishes
   3. Needs
9. Goals achieved
   1. Check flower association
