# Supplementary figures and images for: Mobile Health Apps for the Control and Self-management of Type 2 Diabetes Mellitus: Qualitative Study on Users’ Acceptability and Acceptance
Source: JMIR Diabetes. 2023 Jan 24;8:e41076. doi: 10.2196/41076 (PMC9947812; doi:10.2196/41076)

**Multimedia Appendix 4. Coding tree**


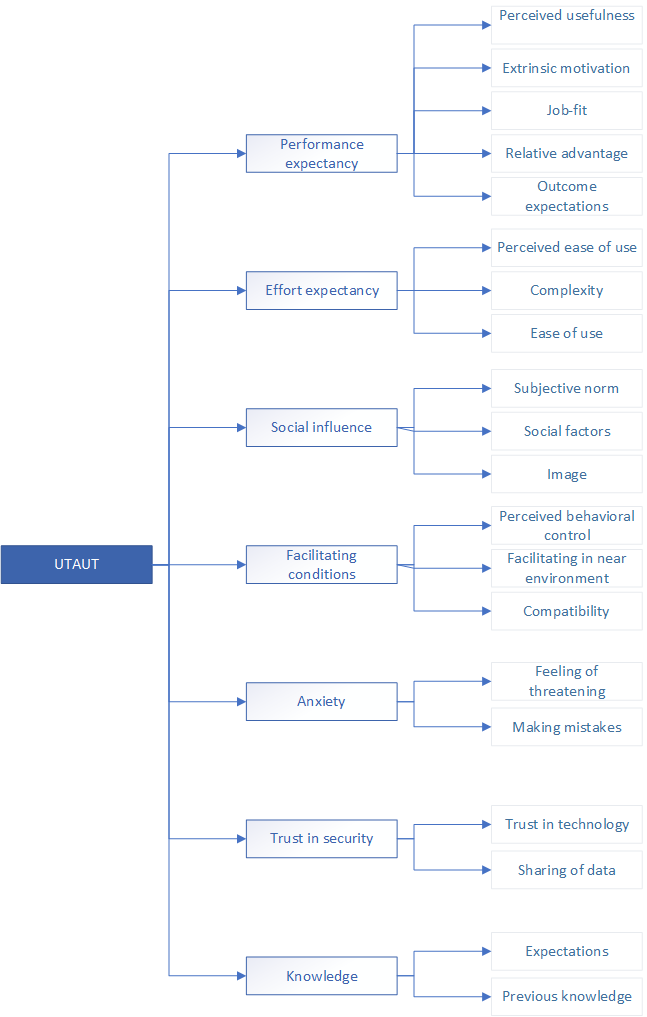

Supplement: Multimedia Appendix 4 [file diabetes_v8i1e41076_app4.docx]
